# Contamination Study of Zirconia on the Densification Process and Properties of Transparent MgAl_2_O_4_ Ceramics

**DOI:** 10.3390/ma12050749

**Published:** 2019-03-05

**Authors:** Yi Li, Qun Zeng, Jian Zhang, Dan Han, Shiwei Wang

**Affiliations:** 1 Guangzhou Key Laboratory for Special Fiber Photonic Devices and Applications, School of Information and Optoelectronic Science and Engineering, South China Normal University, Guangzhou 510006, China; liyiliyi7@163.com; 2 State Key Laboratory of High Performance Ceramics and Superfine Microstructure, Shanghai Institute of Ceramics, Chinese Academy of Sciences, Shanghai 200050, China; handan@mail.sic.ac.cn (D.H.); swwang51@mail.sic.ac.cn (S.W.); 3 Key Laboratory of Transparent Opto-functional Inorganic Materials, Shanghai Institute of Ceramics, Chinese Academy of Sciences, Shanghai 201899, China; 4 Center of Materials Science and Optoelectronics Engineering, University of Chinese Academy of Sciences, Beijing 100049, China

**Keywords:** spinel, transparent ceramics, zirconia, microstructure, optical properties

## Abstract

In this paper, transparent magnesium aluminate (MgAl_2_O_4_) spinel ceramics are fabricated through pressureless sintering combined with hot isostatic pressing (HIPing). To investigate the effect of zirconia on sintering behavior, microstructure, and optical properties of transparent spinel ceramics, different contents of zirconia were added. The results show that zirconia can promote the densification process by the formation of anion vacancies. The resulting zirconia exists as tetragonal phase along the grain boundaries. The average grain size of resulting ceramics depends on the content of zirconia and HIPing condition. Small zirconia content did not deteriorate the optical properties of samples. A 5-mm-thick sample with 0.05 wt% ZrO_2_ pre-sintered at 1500 °C followed by HIPing treatment at 1550 °C achieved a high in-line transmittance of 75.5% at 400 nm and 85% at 1100 nm.

## 1. Introduction

Transparent magnesium aluminate spinel ceramics (hereafter termed “spinel”) have been well studied for more than 50 years, since the first translucent sample was successfully fabricated in the 1960s [1,2]. With the advantages of high hardness, high flexure strength, excellent transparency over 0.2–6 μm [3,4,5,6], spinels are widely used as protective materials in applications such as transparent armors, temperature viewing windows, and IR domes for IR seekers [6,7,8,9].

To obtain fully dense transparent spinel ceramics, the residual porosity in the sintered sample should be carefully controlled. The pore diameter of resulting samples should be under 40 nm, which markedly decreases the scattering in the visible range. Additionally, the amount of pores should be less than 100 ppm according to Mie-scattering theory [10,11,12,13]. Reducing the porosity to an acceptable level is the main challenge for the fabrication of transparent spinel ceramics. The spinel densification rate is mainly dominated by O^2−^ diffusivity, as it is the largest ion and close-packed lattice [3,10,14,15]. Due to the low diffusivity of O^2−^ in spinel structure, extreme sintering conditions of high temperature and high pressure are usually required to achieve full densification. High sintering temperature is normally accompanied by rapid grain growth, which results in low mechanical properties. At present, the key method to reduce sintering temperature is associated with nanopowder with high sintering ability [16]. However, most nanopowders face the problem of agglomeration, which is harmful to the homogeneity of green body and the following densification process [17]. When using nanopowder as the starting material, mechanical milling is necessary for removing agglomerates. High-quality zirconia ball is one of the most common milling media to fabricate large-sized transparent ceramics. In transparent ceramics, it is extremely important to control the contamination introduced by the preparation process. However, it is inevitable that the milling loss from the zirconia ball will be introduced into high-purity spinel powder. The introduced contamination could affect the sintering behavior, microstructure, and optical and mechanical properties of transparent spinel ceramics [18].

As reported previously, some transition-metal cations are soluble and can incorporate into the lattice, affecting the diffusion and mechanical properties by creating defects [19,20]. Zirconia was commonly used as sintering additive in the MgAl_2_O_4_ type of refractory material. The resulting samples containing zirconia (0.5–2 wt%) exhibited higher flexural strength [21]. The mechanism by which ZrO_2_ promoted densification was demonstrated by Kim [22].The reaction between MgO and ZrO_2_ can enhance the cation diffusion. Nevertheless, the research on zirconia in the fabrication of transparent ceramics was rare. G. Bernard-Granger et al. chose the combination of TiO_2_ and ZrO_2_ (stabilized with 3 mol% of yttrium oxide) with tens of ppm atomic as sintering additive to fabricate transparent spinel ceramics [23]. The in-line transmittance of resulting samples were significantly improved from 68.1% to 82.3% at 300 nm. Meanwhile, the flexural strength of resulting samples were enhanced from 240 to 330 MPa. However, the mechanism of co-dopants acting in the sintering process is not mentioned. Therefore, the role of zirconia in preparing transparent spinel ceramics still needs further research.

In this paper, different contents of monoclinic zirconia are added into commercial MgAl_2_O_4_ nanopowder to fabricate transparent ceramics. The effect of zirconia on the sintering process, microstructure evolution, and optical and mechanical properties are investigated in detail. Further, the maximum tolerance of transparent spinel ceramics to zirconia contamination can be determined.

## 2. Materials and Methods

### 2.1. Experimental Procedure

High-purity commercial MgAl_2_O_4_ spinel nanopowder (S30CR, Baikowski, France) was selected as the starting material. The primary particle size was about 55 nm. Agglomerates were mainly exceeding 1 μm. Monoclinic zirconia powder (G0Y-010OO, Shandong Sinocera Functional Material Co., Ltd, Dongying, China) with average particle size of ~90 nm was selected as the zirconium source. Agglomerates were ~140 nm. Into the spinel powder, 0, 0.01, 0.05, 0.1, and 1 wt% monoclinic zirconia powder were added, marked as A to E, respectively. Spinel powder with 5 wt% zirconia was prepared for further testing to investigate the possible reaction between spinel powder and zirconia at elevated temperature. The raw material without and with various contents of zirconia were mixed by ball-milling in a nylon jar with alumina balls at 250 rpm/h for 12 h. The mixed slurry was dried at 60 °C for 24 h in an oven. Then, the dried powder was sieved through a 100-mesh screen and calcined at 800 °C for 6 h to remove organic impurities. The green bodies were shaped by cold biaxial pressing at 20 MPa, followed by cold isostatic pressing for 5 min under 200 MPa pressure. Next, the green bodies were pre-sintered in a muffle furnace in air for 3 h in the temperature range of 1450–1550 °C. In order to eliminate the residual closed pores in the pre-sintered samples, the samples without open pores (marked as 1 to 4, representing the pre-sintering temperatures of 1500, 1515, 1525, and 1550 °C, respectively) were hot isostatic pressed (HIPed) at 1550 °C and 190 MPa for 3 h in argon atmosphere. For the further tests, the resulting samples were ground and mirror-polished on both sides to 5 mm thick.

### 2.2. Characterization

The Archimedes principle was employed to calculate the relative density and open porosity of pre-sintered samples. The wet weight and buoyant weight of samples were measured after boiling to ensure open pores were filled with water. The microstructure of the pre-sintered and HIPed samples was characterized by scanning electron microscopy (SEM, JSM-6390, JEOL, Tokyo, Japan), and a field emission scanning electron microscope (FE-SEM, SU8220, Hitachi, Japan) with an energy dispersive spectrometer (SwiftED3000, Hitachi, Tokyo, Japan). The average grain size ($\bar{\mathrm{GS}}$) of the pre-sintered and HIPed samples was evaluated by linear intercept analysis according to the equation $\bar{\mathrm{GS}}=1.56\;\bar{L}$, where $\bar{L}$ is the mean intercept. More than 200 grains were taken into account. The in-line transmittance of transparent samples was measured by a UV–VIS–NIR spectrometer (Carry 5000 spectrophotometer, Varian, Seattle, USA) in the range of 200–1100 nm. The measurements of Vickers hardness and fracture toughness of HIPed samples were performed by indentation method on a Vickers hardness instrument (TUKON-2100B, Instron Co., Norwood, MA, USA) with a load of 29.4 and 9.8 N, respectively. Each sample was measured five times on different areas of the polished surface. The calculation of the fracture toughness was based on the length of cracks and indentations [24]. The crystalline phase of mixed powder calcined from 1150 to 1500 °C was tested by X-ray diffraction (D8, Bruker, Berlin, Germany) using CuKα radiation (k = 1.5406 Å) at 40 kV and 100 mA.

## 3. Results

### 3.1. Pre-Sintering Process

**Figure 1:**
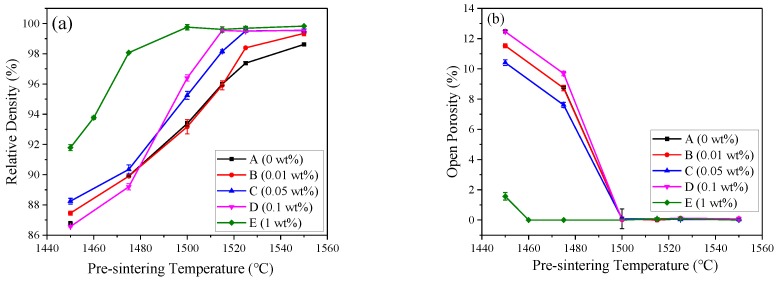
Densification process of samples A to E pre-sintered in the range of 1450–1550 °C: (**a**) relative density with respect to the pre-sintering temperature; (**b**) open porosity versus the pre-sintering temperature.

In order to investigate the effect of ZrO_2_ on the densification process, samples with different contents of ZrO_2_ were pre-sintered at temperatures from 1450 to 1550 °C. The relative density and open porosity of pre-sintered samples are summarized in Figure 1a,b, respectively. The densification process was enhanced through the increase of the pre-sintered temperature (Figure 1a). Sample A achieved the relative density of 86.80% at 1450 °C. The relative density of sample A gradually increased with the pre-sintering temperature and reached 98.62% at 1550 °C. The rapid densification process happened from 1460 °C. Compared with sample A, the densification process of sample B was almost the same, while that of sample C was faster. When the content of zirconia increased to 1 wt%, the densification process was significantly improved. The relative density of sample E pre-sintered at 1450 °C was 91.79%. Moreover, it is surprising that the required pre-sintered temperature of sample E to achieve near-theoretical density was 1500 °C, which was at least 20 °C lower than that of the sample A. To carry out the hot isostatic pressing (HIPing) treatment, the open porosity in pre-sintered samples should be eliminated to zero. As shown in Figure 1b, the open porosity of sample E was totally eliminated at 1460 °C, while other samples required higher temperature to achieve this level. It is indicated that zirconia showed a beneficial effect in promoting densification, reducing the required pre-sintering temperature and open porosity before HIPing treatment.

**Figure 2:**
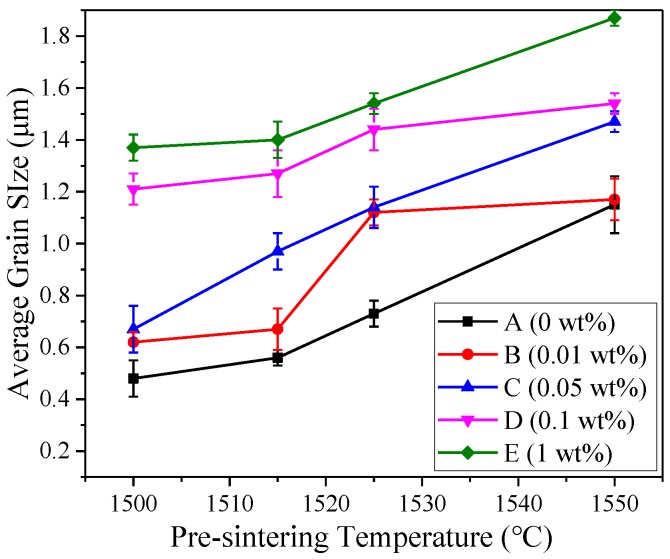
Pre-sintering behavior during pressureless sintering in air: average grain size versus pre-sintering temperature.

The average grain size of samples A to E pre-sintered at various temperature is presented in Figure 2. In general, five batches of pre-sintered samples exhibited the same tendency. The grain growth happened with the increase of temperature. The average grain size of sample A1 reached 0.48 μm at 1500 °C. When the content of zirconia increased to 0.05 wt%, the average grain size of sample C1 showed a slight increase at the same pre-sintering temperature. The grain sizes of samples D1 and E1 were about twice as large as the others. At 1550 °C, the average grain size of all samples was much larger than those pre-sintered at lower temperature. It can be concluded that zirconia can obviously accelerate grain growth during the pre-sintering process.

### 3.2. Properties and Microstructure of Transparent Spinel Ceramics

#### 3.2.1. Optical Transparency

**Figure 3:**
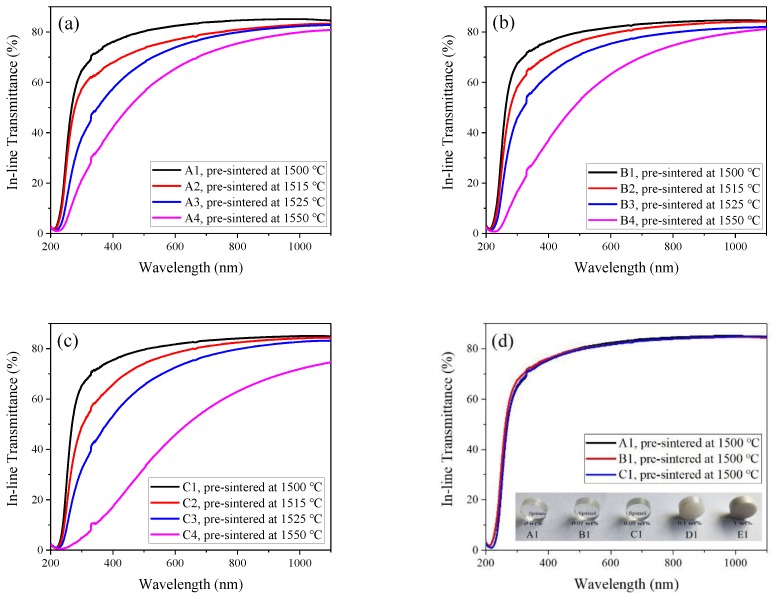
In-line transmittance of 1550 °C HIPed samples as a function of pre-sintering temperature (Numbers 1–4 represent samples pre-sintered at 1500, 1515, 1525, and 1550 °C, respectively): (**a**) A; (**b**) B; (**c**) C; (**d**) optimal in-line transmittance with respect to different contents of zirconia. The inset pictures show the appearance of samples A1 to E1 from left to right, respectively. All samples are 14 mm in diameter and 5 mm in thickness.

Pre-sintered samples without open pores were selected for HIPing treatment at 1550 °C. Samples of batches D and E were opaque after HIPing treatment. The in-line transmittance curves are presented in Figure 3. As shown in Figure 3a–c, the in-line transmittance of all samples decreased with the increase of pre-sintering temperature, which is consistent with the results reported by Goldstein et al. and others [25,26,27]. For each batch, samples with optimal in-line transmittance were obtained by lower pre-sintering temperature. This phenomenon was probably caused by tiny intragranular pores produced by the rapid densification process at high temperature. However, intragranular pores cannot be eliminated during HIPing treatment, and deteriorate the in-line transmittance in the range of 200–600 nm [10]. Hence, the pre-sintering process should focus on preparing pre-sintered samples with proper grain size and homogeneous microstructure for the following HIPing treatment. With the proper control of pre-sintered and HIPed condition, samples containing zirconia up to 0.05 wt% exhibited no obvious distinction in optical transparency compared with samples without zirconia (Figure 3d). Sample C1 achieved a high in-line transmittance of 75.5% at 400 nm and 85% at 1100 nm, which was nearly equal to the in-line transmittance of sample A1.

#### 3.2.2. Microstructures

**Figure 4:**
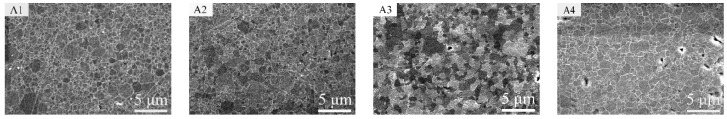

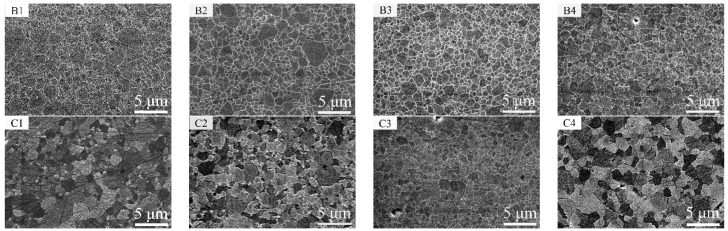
Microstructure of samples A, B, and C pre-sintered at different temperatures (1, 1500 °C; 2, 1515 °C; 3, 1525 °C; 4, 1550 °C) and HIPed at 1550 °C.

The thermal etched surfaces of the HIPed samples were characterized by SEM (Figure 4). The results confirmed that the microstructure of all HIPed samples was homogeneous. No residual pores were observed along grain boundaries. With the increase of zirconia, the average grain size of HIPed samples A1, B1, and C1 (listed in Table 1) increased slightly. This is quite different from the Zr^4+^ doped yttrium aluminum garnet (YAG) ceramic, where Zr^4+^ can inhibit grain growth in YAG [28,29]. The mechanism of zirconia promoting grain growth in the spinel system needs further research. No exaggerated grain was observed in SEM images of all samples, which was usually observed in samples containing LiF additive [3,19]. When increasing the HIPed temperature to 1600 °C, samples A to C exhibited a bi-modal grain size distribution. This was shown in a previous report as well [30]. Besides, for each batch, regardless of the difference in grain size of pre-sintered samples, the resulting grain size of HIPed samples was nearly equal. It was clearly revealed that the resulting grain size of HIPed samples depends on the HIPing condition and zirconia rather than the pre-sintering condition.

### 3.3. Phase Analysis

**Figure 5:**
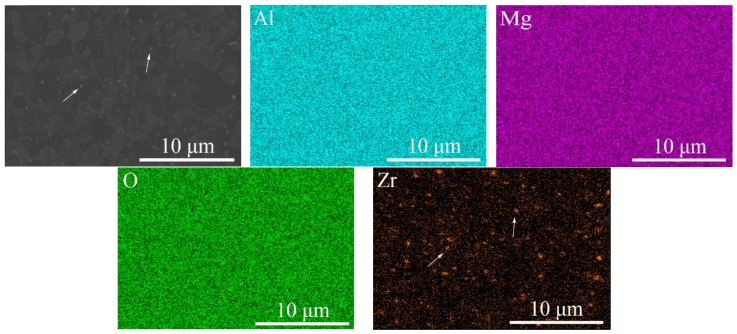
SEM/EDS mapping analysis of opaque sample E.

To determine the distribution of zirconia in the samples, the microstructure of opaque sample E pre-sintered at 1475 °C and HIPed at 1550 °C was characterized by a field emission scanning electron microscope with an energy-dispersive spectrometer. For precise detection, the thermally etched sample was coated with carbon instead of Au to avoid characteristic peak overlap. The result (Figure 5) showed that white spots were randomly located along the grain boundaries, which was consistent with a previous work reported in refractory material [21]. According to EDS mapping analysis, the white spots contained zirconium. With the mismatch of refractive index between zirconia and MgAl_2_O_4_, the random distribution of zirconia in samples acted as scattering centers, which was detrimental to the in-line transmittance. Besides, the grain size of resulting zirconia was about 300 nm, which is also harmful to transparency according to scattering theory. This was why the samples of batches D and E were opaque.

**Figure 6:**
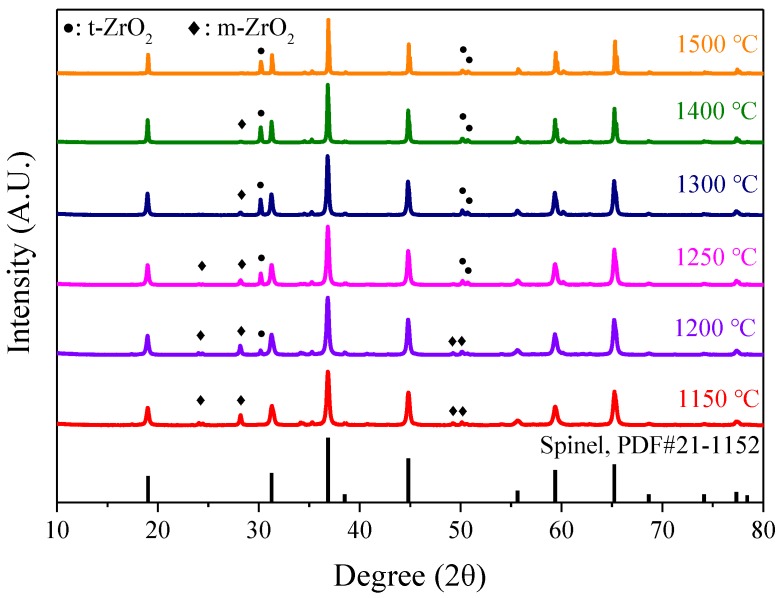
XRD patterns of spinel powder with 5 wt% ZrO_2_ as a function of calcining temperature.

With the intent of better understanding the role of zirconia in the sintering process, a relatively large proportion of zirconia (5 wt%) was mixed with spinel powder and calcined at different temperatures to detect any reaction products more easily. The calcined powders were characterized by X-ray diffraction patterns (Figure 6). According to the phase diagram of binary MgO–ZrO_2_, MgO could react with pure zirconia above 1150 °C, acting as the stabilizer, to stabilize tetragonal zirconia at room temperature [31,32]. However, Mg^2+^ locates in a tetrahedron site in the lattice of MgAl_2_O_4_. To obtain Mg^2+^ from the spinel lattice for zirconia, maintaining the tetragonal phase must be more difficult. As shown in Figure 6, zirconia existed as monoclinic phase below 1150 °C. The tetragonal phase started forming at 1200 °C. With the increase of calcining temperature, the content of monoclinic zirconia decreased, while the tendency of the content of tetragonal zirconia was opposite. When the calcining temperature increased to 1400 °C, the characteristic peaks of monoclinic zirconia almost disappeared. This suggested that zirconia can react with MgAl_2_O_4_ during the sintering process. The most possible mechanism was that, with similar ionic radius, Mg^2+^ precipitated from the spinel lattice and incorporated into ZrO_2_, playing the role of stabilizing the zirconia as a tetragonal phase. As for spinel, the formation of anion vacancies ($V_{\mathrm{Mg}}^{\prime \prime }$) accelerated mass transfer and promoted the densification process during the sintering process. Besides, compared to monoclinic zirconia, the stabilized zirconia prohibited the formation of cracks during the cooling process, which could be beneficial for the mechanical properties of the samples.

### 3.4. Hardness and Fracture Toughness

The Vickers hardness and fracture toughness of samples with optimal in-line transmittance were measured, and values are shown in Table 1. The Vickers hardness of samples had a small decrease with the increasing content of zirconia, which was associated with grain growth [33]. The fracture toughness of samples with zirconia was nearly equal to samples without zirconia. Hence, zirconia does not significantly impact the mechanical properties of the resulting ceramics.

## 4. Conclusions

Transparent MgAl_2_O_4_ ceramics with and without zirconia were successfully fabricated through pressureless sintering followed by HIPing treatment. The results showed that zirconia can promote the densification process by producing anion vacancies in the spinel lattice, accelerating mass transfer. Tetragonal zirconia was formed during the sintering process because Mg^2+^ can incorporate into ZrO_2_. With the presence of zirconia, the average grain size of pre-sintering samples was significantly affected. The final grain size of HIPed ceramics was determined by zirconia and HIPing condition. Samples with 0.1 wt% and 1 wt% ZrO_2_ were opaque because zirconia was randomly located at the grain boundaries, acting as scattering centers. Hence, the degree of zirconia contamination should be controlled at less than 0.05 wt%, which demonstrated no obvious influence on optical or mechanical properties. With proper control of milling loss, it is possible to use zirconia ball as milling media to de-agglomerate nanopowder for the fabrication of large-sized transparent spinel ceramics.

## Figures and Tables

**Table 1:** Vickers hardness and fracture toughness of samples with optimal in-line transmittance.

Sample	Content of Zirconia (wt%)	Average Grain Size of HIPed Sample (μm)	Pre-Sintering Temperature (°C)	Vickers Hardness (GPa)	$\mathrm{Fracture}\;\mathrm{Toughness}\;(\mathrm{MPa}\sqrt{m})$
A1	0	1.3	1500	14.47 ± 0.14	1.41 ± 0.04
B1	0.01	1.7	1500	14.23 ± 0.37	1.47 ± 0.04
C1	0.05	2.0	1500	14.18 ± 0.15	1.43 ± 0.04
